# B lymphocytes play a limited role in clearance of *Campylobacter jejuni* from the chicken intestinal tract

**DOI:** 10.1038/srep45090

**Published:** 2017-03-23

**Authors:** Lizeth Lacharme-Lora, Gemma Chaloner, Rachel Gilroy, Suzanne Humphrey, Kirsty Gibbs, Sue Jopson, Elli Wright, William Reid, Julian Ketley, Tom Humphrey, Nicola Williams, Steven Rushton, Paul Wigley

**Affiliations:** 1 Institute for Infection and Global Health and School of Veterinary Science, University of Liverpool. Leahurst Campus, Neston, CH64 7TE, UK; 2 Institute for Research on the Environment and Sustainability, Newcastle University, Newcastle upon Tyne, NE1 7RU, UK; 3 Department of Genetics, University of Leicester, Leicester LE1 7RH, UK; 4 Medical School, Swansea University, Swansea SA2 8PP, UK

## Abstract

*Campylobacter jejuni* is the leading cause of foodborne bacterial gastroenteritis with contaminated poultry meat its main source. Control of *C. jejuni* is a priority for the poultry industry but no vaccines are available and their development hampered by poor understanding of the immunobiology of *C. jejuni* infection. Here we show the functional role of B lymphocytes in response to *C. jejuni* in the chicken through depletion of the B lymphocyte population (bursectomy) followed by challenge. B lymphocyte depletion has little effect on bacterial numbers in the ceca, the main site of colonisation, where *C. jejuni* persist to beyond commercial slaughter age, but reduces clearance from the small intestine. In longer-term experiments we show antibody leads to reduction in *C. jeuni* numbers in the ceca by nine weeks post infection. Whilst we did not examine any protective role to re-challenge, it illustrates the difficulty in producing a vaccine in a young, immunologically naïve host. We believe this is first study of functional immunity to *C. jejuni* in chicken and shows antibody is ineffective in clearing *C. jejuni* from the ceca within the production lifetime of chickens, although is involved in clearance from the small intestine and longer-term clearance from the ceca.

*Campylobacter jejuni* is the most common cause of foodborne bacterial gastroenteritis worldwide^1^. Chicken is the most frequent source of human infection and as such, control of infection in poultry production is a public health priority^2^. Effective vaccination, which has proved successful for the control of *Salmonella enterica* in chicken and egg production offers considerable long-term potential in controlling *C. jejuni,* but vaccine development has been hampered by a relatively poor understanding of the infection biology of, and in particular the immune response to, *C. jejuni* in the chicken^3^.

*C. jejuni* is able to colonise the intestinal tract, and in particular the large blind ceca at the junction of the small and large intestines to a high level, with bacterial counts of 10^8^ CFU per gram or even higher frequently found^3^. Although often considered to be a commensal organism ‘tolerated’ by the chicken immune system^4^, we have recently shown that there is an initial inflammatory response to *C. jejuni* infection in the chicken intestine that can lead to pathology and diarrheal disease^5^. Adaptive responses to *C. jejuni* are poorly described in the literature, and there is a lack of functional studies of adaptive immunity in the chicken. Cytokine responses that drive cellular, humoral and Th17 responses are found in the gut following infection^6^,^7^,^8^, and antibody responses to infection and specific antigens including LPS, LOS and flagella have been described previously^9^,^10^,^11^. Furthermore, various experimental vaccines have produced specific antibody responses, although none have been found to be fully protective or able to elicit protection via a strategy that is cost effective for the poultry industry^12^,^13^,^14^,^15^,^16^. Maternally-derived antibodies are thought to offer a degree of protection because in the field, birds are rarely colonised in the first two weeks of life and this ‘lag phase’ of colonisation correlates well with the decline in specific maternally-derived antibodies in the chick^17^. However, as yet there are no truly functional studies that confirm any protective role.

It is challenging to perform functional immunological studies in non-biomedical species, as there is not an array of readily-available functional genetic knockouts for livestock species as there are for mice. The use of transgenic chickens in experimental studies is very much in its infancy and certainly not available for the rapid-growing broiler chicken breeds used in production. However, a distinct divergent evolutionary feature of the avian immune system, the Bursa of Fabricius, allows functional studies of antibody and B lymphocytes to be made through bursectomy, the removal of the organ through surgery or ablation via chemical or hormonal treatment. Bursectomy gives rise to birds with a highly depleted B lymphocyte population. In this study we have utilised cyclophosphamide treatment of newly-hatched commercial broiler chicks to deplete the B lymphocyte population and to determine the role antibody plays in limiting colonisation and in clearance of *C. jejuni* from the broiler chicken intestinal tract.

## Results

**Figure 1:**
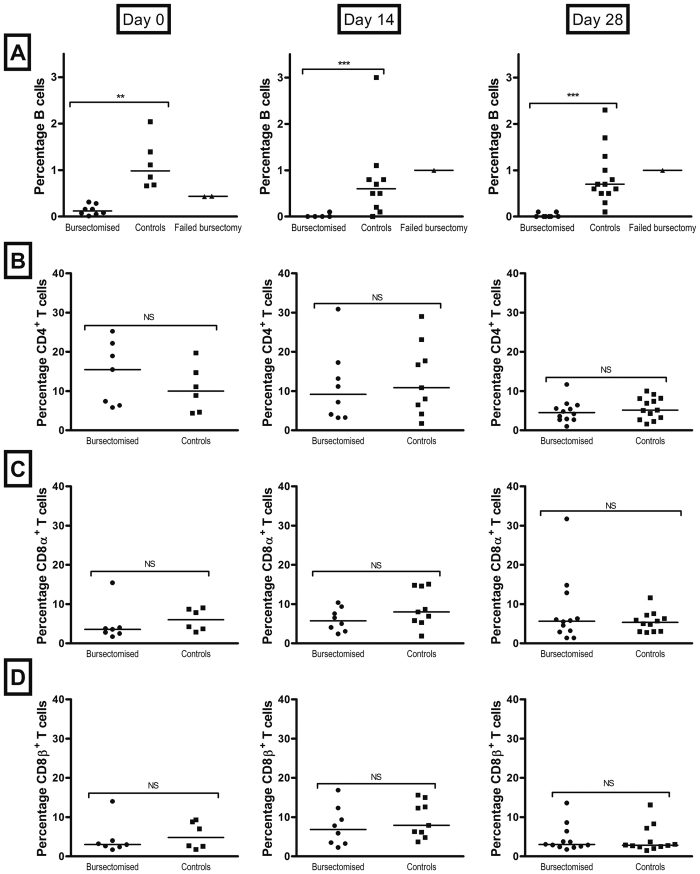
Effect of bursectomy on B and T cell populations. B cell depletion in young chicks was achieved by the chemical bursectomy method using cyclophosphamide. To assess the effect of the treatment on B and T cell populations, blood samples from birds were taken at 3, 5 and 7 weeks of age (day 0 and 14 and 28 days post infection respectively). The Bu-1 marker was used to identify avian B lymphocytes (**A**) and CD4, CDα and CD8β markers were used for T lymphocytes (**B**–**D**). Bars represent the median values; circles represent individual birds. At each time point there were 10–12 birds per group. Statistically significant differences were only observed in the B cell population. **p ≤ 0.01; ***p ≤ 0.001; NS = non-significant.

Bursectomy of birds using cyclophosphamide resulted in a marked reduction in the size of the bursa and a depletion of more than 90% of the bursal B cell population 3 weeks after treatment. The circulating B cell population further decreased after 5 and 7 weeks (14 and 28 days post infection, Fig. 1A). Control birds had a significantly higher level of B cells than bursectomised birds (t = 8.439, P = 2 × 10^−11^), and this did not change with time (t = −1.349, P = 0.1852). The proportion of CD4^+^ cells was not effected by bursectomy (t = 0.612, P = 0.543), but there was a significant decline in CD4^+^ cells present with time in both control and treated birds (t = −3.569, P = 0.0008). Levels of CD8α^+^ and CD8β^+^ cells were not different between treated and control birds (t = 1.72, P = 0.092 and t = 1.338, P = 0.187 respectively), neither was there any trend with time (t = 0.428, P = 0.671 and t = −0.762, P = 0.45, respectively).

There was no effect of bursectomy on T cell population evident at any time point (Fig. 1B–D). In a few cases the bursectomy procedure failed and the B cell population and subsequent production of antibody from these birds was similar to that of the controls (Fig. 1A). Bursectomy had no discernible effect on bird health, weight gain or susceptibility to other infection through the course of the experiment, though as housed under high biosecurity conditions there is no potential exposure to infectious agents (supplementary information).

### The B lymphocyte population plays a limited role in reducing colonisation of the ceca by *C. jejuni* but is associated with reduction in colonisation of the small intestine

**Figure 2:**
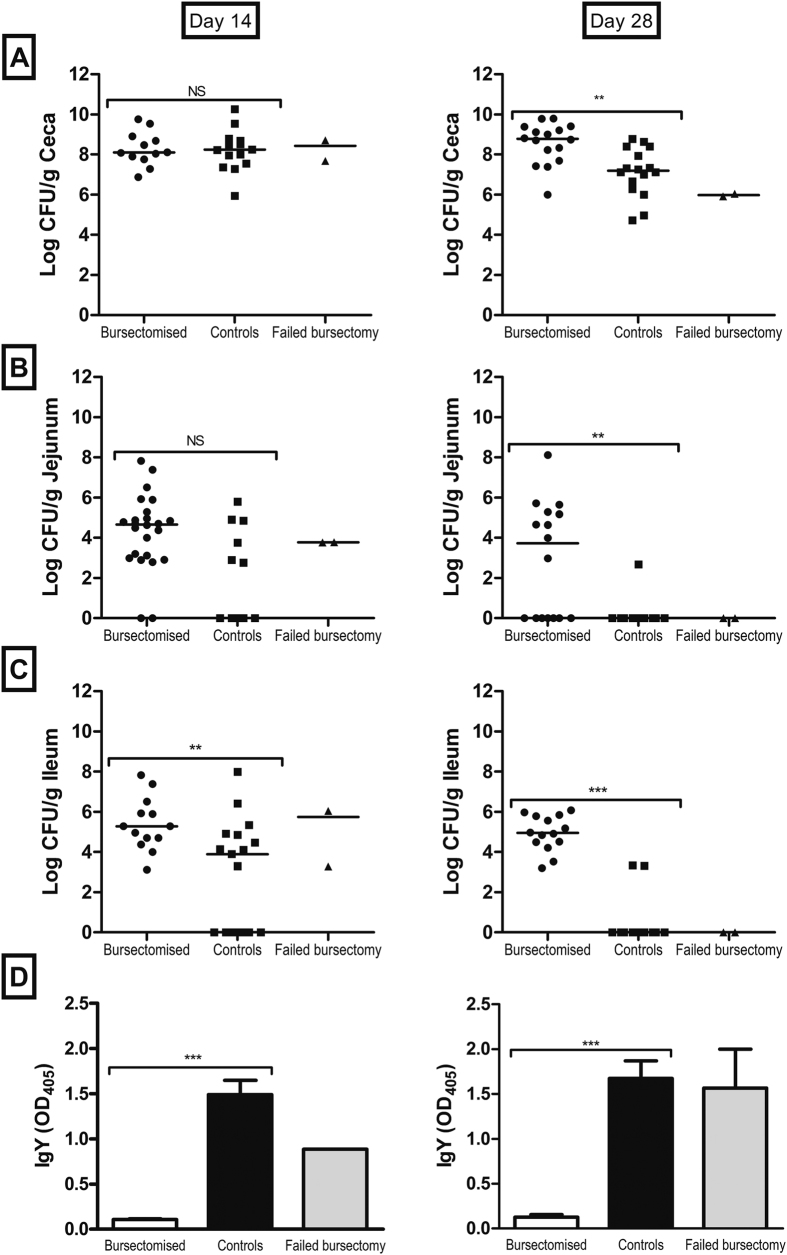
Colonisation of the chicken intestinal tract by *C. jejuni* (**A**–**C**) and specific antibody levels in serum (**D**) at 14 and 28 days post infection. Colonisation was examined in the ceca, jejunum and ileum and is presented as the CFU of *C. jejuni* per gram of gastrointestinal content. Levels of specific IgY antibodies against *C. jejuni* in serum were determined at each time point. Birds were challenged with *C. jejuni* M1 at 3 weeks of age. At each time point there were 10–12 birds per group. Bars represent the median values; circles represent individual birds. **p value ≤ 0.01; ***p value ≤ 0.001; NS = non-significant.

To study the role of B lymphocytes and antibody in controlling intestinal colonization by *C. jejuni* we challenged bursectomised and control birds with *C. jejuni* M1 isolate and followed colonisation and antibody levels for up to 28 days post infection. At 14 days post-infection, high titres of anti-*Campylobacter* antibodies were present in the serum of control birds. Bursectomised birds failed to produce specific circulating anti-*C jejuni* IgY (Fig. 2D) or IgM (not shown) antibodies in response to infection. In regard to colonisation of the ceca, both groups of birds showed high levels of cecal colonisation at 14 and 28 days post infection, regardless of the presence of antibodies in serum (Fig. 2A). Nonetheless, colonisation levels in control birds were lower at 28 days post infection, suggesting that antibody could potentially play a role in the eventual clearance of *C. jejuni.* Importantly, our data at 14 and 28 days post infection indicate that antibody has a distinct effect in the small intestine, where *C. jejuni* is cleared from the jejunum and ileum of most of the control birds by 28 days post infection This was also observed in birds where the bursectomy procedure failed (Fig. 2B,C).

Bursectomised birds therefore had on average a 19 fold higher level of *Campylobacter* counts than non-bursectomised (t = 2.464 P = 0.01446). These levels increased down the intestinal tract from the jejunum to ceca (t = 17.07, P = 2 × 10^−16^). However, the bacterial count declined with time from colonisation (t = −5.638, P = 5.07 × 10^−8^). There was also a significant interaction between whether or not a bird was bursectomised and the decline through time, with bursectomised birds generally having a higher count across all gut regions as time passed (t = 3.024, P = 0.00278). The results indicate that bursectomy allows greater levels of colonisation by *C. jejuni,* with all regions of the gut having higher loads than control birds. Whilst *C. jejuni* loads declined through time across both treated and untreated birds the decline in bacterial numbers was lower in bursectomised birds.

We used structural equation modelling to examine the effect of bursectomy on the production of IgY and in turn whether this influenced the colonisation of *C. jejuni*. We examined three different models of colonisation using broilers that had either been bursectomised or not. These were: 1) *C. jejuni* colonises the ileum first and then spreads to the jejunum and caecum; 2) *C. jejuni* colonises the caecum and then spreads to the ileum followed by the jejunum; and 3) *C. jejuni* colonises the jejunum and spreads down the gastrointestinal tract to the ileum and then caecum. For each model we examined the following goodness of fit statistics: 1) χ^2^-test, where a Bollen-Stine adjusted *P* > 0.05 indicates that the observed and expected covariance matrices for the model are not different; 2) the root mean square approximation (RMSEA) < 0.05 and 90% confidence intervals; 3) standardised root mean square residuals (SRMR) < 0.08; and 4) comparative fit index (CFI) > 0.95^18^,^19^. With the exception of treatment which was categorical (i.e. bursectomised yes or no), all data were log transformed prior to analysis.

**Figure 3:**
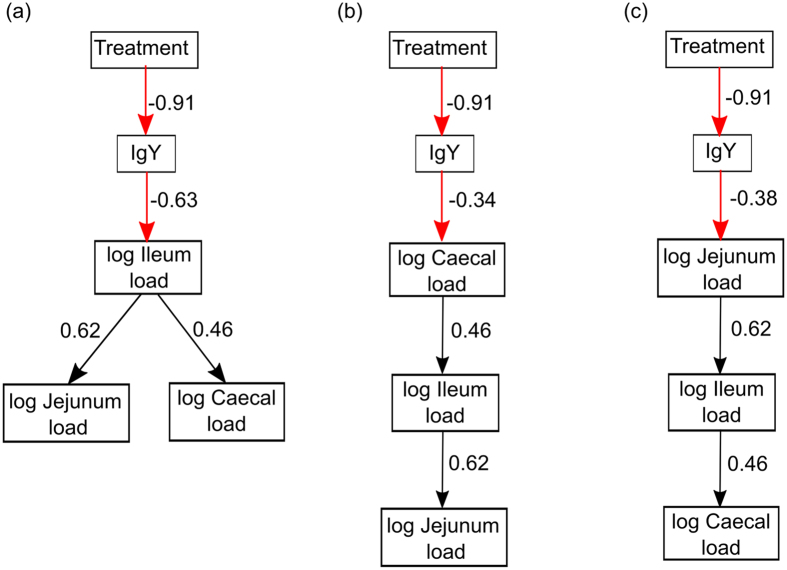
Path diagrams representing the 3 structural equation models describing the influence of IgY on the colonisation of the chicken gut and subsequent spread to other areas of the gastrointestinal tract. The standardised path coefficients for each response are shown on each arrow. The black arrows represent positive relationships and the red arrows represent negative relationships.

The 3 models are presented in Fig. 3. The only one that described the data was model 1, which converged normally after 38 iterations (Fig. 3a) and had the following goodness of fit measures: χ^2^ = 5.126, DF = 5, *P* = 0.612; CFI = 0.999, RMSEA = 0.022, 90% CI = 0.000–0.195; SRMR = 0.043. In models 2 and 3 (Fig. 3b and c), the χ^2^-test indicated a significant difference between the model and the data (*P* < 0.05), the RMSEA scores were >0.200 and the CFI was <0.900. These together suggest that these data could not support models 2 and 3. Model 1 indicated that broilers that had not been bursectomised had greater amounts of IgY (Table 1) and explained a substantial amount of the variance (*R*^2^ = 0.834). The levels of IgY were negatively related to the *C. jejuni* load in the ileum (Table 1) suggesting that there was a link between *C. jejuni* colonisation and immune-competence of the broilers. *C. jejuni* load in the ileum was positively related to *C. jejuni* load in the jejunum and caecum (Table 1). This would indicate that once *C. jejuni* had colonised the ileum it spread outwards to the jejunum and ceca although the former is unlikely as it would require movement against intestinal flow and there are no reports of anti-peristaltic activity associated with any intestinal infection in the chicken. However, the *R*^2^ for ileum, jejunum and caecum were 0.392, 0.386 and 0.214, respectively, indicating that there were other factors controlling the levels of *C. jejuni* in the tissues that were not accounted for the in the model.

### B lymphocytes and antibody plays a role in clearance of *C. jejuni*

**Figure 4:**
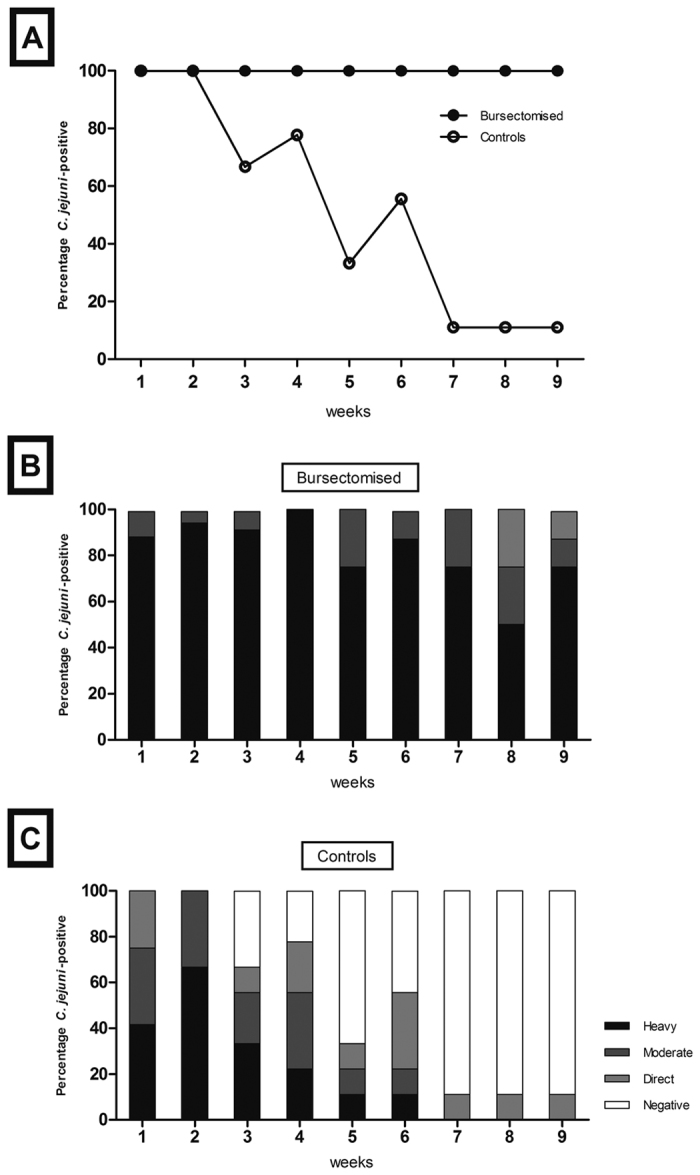
Shedding of *C. jejuni* in bursectomised and control birds. Cloacal swabs from individual birds were taken weekly following challenge with *C. jejuni* M1 at 3 weeks of age (**A**). Shedding was categorised as heavy, moderate, low or negative (**B** and **C**). Data is presented as percentage of birds *Campylobacter*-positive per shedding category based on eight to ten birds per group at each time point.

**Figure 5:**
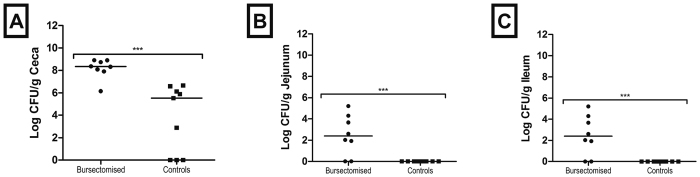
Colonisation of the intestinal tract 63 days post infection. Colonisation was examined in the ceca, jejunum and ileum and is presented as the CFU of *C. jejuni* per gram of gastrointestinal content. Birds were challenged with *C. jejuni* M1 at 3 weeks of age. There were 8 birds in the bursectomised group and 9 birds in the control group at the end of the experiment. Bars represent the median values; circles represent individual birds. **p ≤ 0.01; ***p ≤ 0.001.

**Figure 6:**
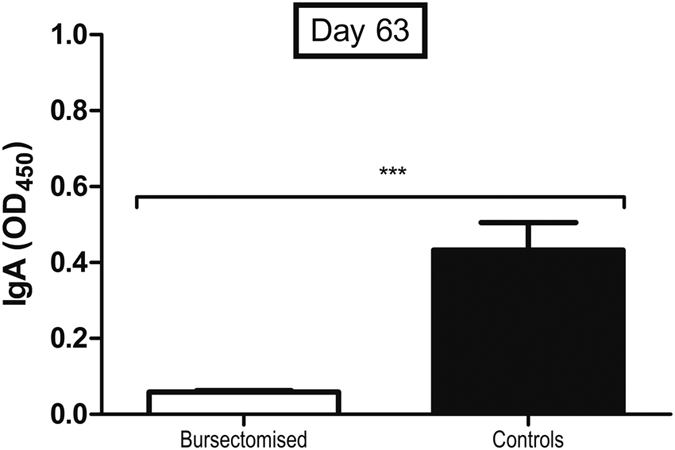
Total secretory IgA in the chicken ileum 63 days post infection. Ileal wash samples were collected from birds were challenged with *C. jejuni* M1 at 3 weeks of age. There were 8 birds in the bursectomised group and 9 birds in the control group.

Although cecal colonisation was not significantly reduced by the presence of antibody at 14 days post infection, there was a trend towards reduction at 28 days post infection and there was a clear reduction in colonisation of the small intestine. This led us to hypothesize that antibody could play a role in clearance. To assess this, we challenged control and bursectomised birds with *C. jejuni* and followed them for up to 9 weeks post infection (84 days of age). We determined *C. jejuni* fecal shedding by taking weekly cloacal swabs and found that shedding levels were decreased in control birds, but there was no change in the bacterial numbers in bursectomised chickens. Semi-quantitative assessment of cloacal shedding in the two groups of birds indicates that the presence of antibody leads to lower levels of *C. jejuni* shed from the cloaca, while in the absence of antibody, high levels of shedding were consistently found throughout the 9-week period (Fig. 4). When we examined the intestinal contents of these birds *post mortem* we found that control, but not bursectomised birds, were largely clear of *C. jejuni* from the small intestine and there was a >2 log reduction in cecal colonisation in control birds (Fig. 5). We took samples from intestinal washes to determine the levels of total secretory IgA. High levels of total secretory IgA were observed in the control birds, whereas little or no IgA was produced by bursectomised chickens (Fig. 6).

## Discussion

Taken together, the data show that whilst B lymphocytes play a role in immune clearance of *C. jejuni*, it fails to clear the bacterium from the intestinal tract within the lifetime of a commercial broiler chicken, which is typically around six weeks of age^5^. We show that birds challenged at 21 days of age do not clear infection from their ceca through B lymphocyte-dependent mechanisms by seven weeks of age. We suggest that colonisation starts in the ileum and then spreads to the jejunum and ceca. We believe that the main functional change in bursectomised birds is depletion of the antibody response though we can not definitively rule out that the absence of B cells has other effects such as changes to antigen-presentation. We also present evidence that antibody-mediated immune clearance from the ceca, as determined via reduced faecal shedding and a significant reduction in colonisation levels, occurs at an older age (Figs 4 and 5). However, clearance from the ileum occurs much earlier (Fig. 5). Commercial poultry production utilises breeds of chicken selected for their ability to convert feed to muscle mass quickly and efficiently, and their rapid growth rate masks the fact that chickens are still relatively naïve in terms of mucosal immunity at slaughter age^20^ Whilst the avian innate immune system appears to mature quite rapidly, and responds effectively to challenges with enteric bacterial species such as *C. jejuni* and *Salmonella enterica,* the development of adaptive responses in the gastrointestinal tract is less well understood. Clearance of *S. enterica* from the gut is strongly associated with the development of cell-mediated and antibody responses, although the latter are not required for clearance. However, clearance of *Salmonella* Typhimurium is highly dependent on the age of the birds at infection, with birds older than six weeks clearing infection from the gastrointestinal tract much more efficiently than younger birds^21^. We also see that this is the case for *C. jejuni,* with antibody-associated clearance only becoming apparent after seven weeks of age (Figs 2). Both our findings and those of other authors suggest that the adaptive immune response in the gut only begins to mature at six weeks of age. This could also explain the results of several experimental vaccine studies, where significant levels of protection to *C. jejuni* were only achieved in older chickens^15^,^16^,^17^,^21^,^22^.

Whilst the ceca are considered the main niche for *Campylobacter* colonisation in the chicken gut, some *C. jejuni* are capable of colonising the small intestine. Previous studies have shown that eliciting a strong antibody response does not correlate to increased clearance of, or protection to, *C. jejuni*. However, our model shows that IgY production influences the number of *C. jejuni* in the ileum and that colonisation along the gastrointestinal tract may start at the ileum and spread out to the jejunum and ceca. It is of interest that antibody clears *C. jejuni* from the ileum and jejunum relatively quickly, but not from the ceca. This has implications for vaccine development because it appears that antibody production in the jejunum and ileum does not inhibit the passage of *C. jejuni* bacteria to the more distal ceca and colon, where antibody appears less effective. Instead, antibody production in the ileum and jejunum may stop re-colonization from the ceca, but specific antibody has little effect on clearing *C. jejuni* from the ceca. It may also go some way to explaining why passive transfer of antibody has some effect in reducing colonisation of *C. jejuni*^23^, that this may be a result of preventing passage of the bacteria through the small intestine, rather than having any effect in the ceca.

The use of cyclophosamide depletion of B lymphocytes in commercial chicken breeds has no significant lasting impact on the T lymphocyte population, whereas in the majority of birds, cyclophosamide treatment caused depletion of more than 90% of the circulating B lymphocyte population and even greater deletions in the spleen (Fig. 1, data not shown). The structure and size of the bursa of Fabricius is also greatly depleted, meaning that B lymphocytes are not replaced in circulation after cell death. Birds that have undergone successful bursectomy fail to produce specific antibodies to *C. jeuni* after challenge. This makes cyclophosphamide bursectomy an efficient tool to study functional antibody responses in fast-growing commercial breeds, such as the Ross 308 used here. Nevertheless, in some cases the procedure is not fully effective, leading to a partial or failed bursectomy. In these birds, specific antibody is produced and the pattern of clearance matches that of the control birds. This is not unique to this method, as failure to remove even a small proportion of the bursa by surgical methods can lead to a functional antibody response. It does, however, provide an additional control that shows the differences in colonisation patterns between groups are due to the absence of antibody rather than an effect of reduced T lymphocyte function, albeit in too few animals to allow meaningful statistical analysis.

The findings here have significance for control of *C. jejuni* in the chicken, and in particular for the potential use of vaccination. Firstly, we show that there might be directionality in the way *C. jejuni* colonises the gut, starting at the ileum and then spreading to the jejunum and ceca, and that the production of antibodies plays little role in the clearance of *C. jejuni* in the chicken ceca. Whilst ascending spread from the ileum to the jejunum against intestinal flow seems unlikely, given *C. jejuni* is highly motile that it may have anti-peristaltic activity it is an intriguing possibility that merits more detailed study. Whilst this does not mean that the generation of an appropriate antibody response may not play a role in preventing infection, it does suggest that vaccines designed to primarily elicit humoral responses may be poorly effective in commercial chicken production. Secondly, broiler chickens are immunologically-naïve during rearing, and it is likely to be very challenging to produce protective intestinal immune responses in such animals before slaughter age as is the case for *Salmonella*^20^. Finally, it raises further questions that the immune response in the chicken is one that primarily prevents bacterial spread from the gut to systemic sites, yet is ineffective in clearing the bacteria from the gut. Our previous studies demonstrate the importance of Th17-mediated responses during infection and we propose that the natural immune response in the chicken is one that prevents systemic infection and substantive disease in the bird, but allows colonisation with little long-term ill effects following initial inflammatory diarrhea. That the modelling data also highlights there are multiple pathogen and host factors associated with intestinal colonisation illustrates there are still considerable gaps in our understanding that need to filled to develop controls for *C. jejuni* in poultry.

In summary, we believe this is the first study of functional immunity to *C. jejuni* in the chicken. It demonstrates that antibody production plays a role, albeit limited, in the clearance of intestinal infection. However, immune clearance following infection takes many weeks, allowing long-term persistence in the ceca of birds beyond slaughter age and therefore is likely to have little impact on the risk of foodborne zoonotic transmission from chicken.

## Materials and Methods

### Bacterial strains and culture conditions

*C. jejuni* M1 was grown from stocks maintained at −80 °C on Columbia blood agar (Lab M, Heywood, Lancashire, United Kingdom) supplemented with 5% defibrinated horse blood (Oxoid, Basingstoke, Hampshire, United Kingdom) for 48 h in microaerobic conditions (80% N_2_, 12% CO_2_, 5% O_2_, and 3% H_2_) at 41.5 °C. Liquid cultures were grown for 24 h in 10 ml of Mueller-Hinton broth (MHB) in microaerobic conditions at 41.5 °C and adjusted by dilution in fresh MHB to a final concentration of 10^6^ CFU/ml. All microbiological media were purchased from Lab M Ltd. (Heywood, Lancashire, United Kingdom).

### Experimental animals

All work was conducted in accordance with United Kingdom legislation governing experimental animals under project license PPL 40/3652 and was approved by the University of Liverpool ethical review process prior to the award of the license. All animals were checked a minimum of twice-daily to ensure their health and welfare. Age-matched, 1-day-old Ross 308 broiler chickens of mixed sex were obtained from a commercial hatchery. Chicks were housed in the University of Liverpool high-biosecurity poultry unit. Chicks were maintained separately in equal–sized groups at stocking levels recommended by UK legislation and were given *ad libitum* access to water and a vegetable protein-based diet (SDS, Witham, UK).

All housing and environmental conditions were identical between groups. Given that *C. jejuni* can spread rapidly through groups of co-housed experimental chickens each treatment group needs to be separately housed to accurately assess a single variable^24^. Chicks were housed on wood shavings in floor pens at a temperature of 30 °C, which was reduced to 20 °C when the birds were 3 weeks of age. Prior to experimental infection, all birds were confirmed as *Campylobacter*-free by taking cloacal swabs, which were streaked onto selective blood-free agar (modified charcoal-cefoperazone-deoxycholate agar [mCCDA]) supplemented with *Campylobacter* enrichment supplement (SV59; Mast Group, Bootle, Merseyside, United Kingdom) and grown for 48 h in microaerobic conditions at 41.5 °C. At 21 days of age, birds were orally infected with 10^5^ cells of *C. jejuni* M1 in 0.2 ml of MHB. At 14, 28 or 63 days post challenge birds were culled and postmortem examinations carried out. Bursectomy was achieved by daily intramuscular injection of 3 mg cyclophosphamide (Sigma) during the first 4 days post-hatch.

#### Assessment of *C. jejuni* in intestinal contents

To determine intestinal colonisation samples from ileal, jejunal and cecal contents were collected from individual birds at necropsy, homogenised and diluted in 9 volumes of maximal recovery diluent (MRD). Serial 10-fold dilutions were made of each sample in MRD, and using the method of Miles *et al*.^25^, triplicate 20 μl spots were plated onto mCCDA agar supplemented with SV59. The plates were incubated under microaerobic conditions at 41.5 °C for 48 h, and *Campylobacter* colonies were enumerated to give CFU/g of sample. Liver tissue was also taken to assess extraintestinal spread of *C. jejuni*.

#### Assessment of *C. jejuni* shedding

Enumeration of *C. jejuni* on the cloacal swabs was carried out using a semiquantitative approach^26^. Briefly, cloacal swabs were plated onto mCCDA agar supplemented with SV59. Then, swabs were eluted in 2 ml modified Exeter broth consisting of 1,100 ml nutrient broth, 11 ml lysed defibrinated horse blood (Oxoid, Basingstoke, Hampshire, United Kingdom), *Campylobacter* enrichment supplement SV59 (Mast Diagnostics), and *Campylobacter* growth supplement SV61 (Mast Diagnostics).Then swabs were incubated at 41.5 °C for 48 h and re-plated onto mCCDA agar and incubated for 48 h at 41.5 °C. Plates were scored for the level of bacterial growth as heavy (>50 colonies after initial direct plating), moderate (between 1 and 50 colonies after initial direct plating), low (*C. jejuni* isolated by plating after enrichment in modified Exeter broth for 48 h, or negative after enrichment.

### Fluorescence-activated cell sorting

Lymphocytes were isolated from whole blood using Histopaque. Cell populations were analyzed by fluorescence-activated cell sorting (FACS) on an Accuri C6 Flow Cytometer (BD). Antibodies specific to chicken B cells (Anti-Bu1a + Bu1b antibody [AV20]-FITC; ab24901, Abcam) and T cells (anti-CD4- FITC, 8210-02; anti-CD8α- FITC, 8220-02; and CD8β- FITC,8280-02 from Southern biotech) were used.

#### Determination of antibody responses

Chicken serum IgY levels were determined by ELISA; serum samples were obtained by removing blood from the heart at necropsy. Blood samples were spun at 13000× *g* for 5 min and serum was collected and stored until use. Flat-bottomed 96-well plates were coated with 100 μl/well of *C. jejuni* whole cell lysate antigen in carbonate-bicarbonate buffer (pH 9.6) and incubated overnight at 4 °C. Subsequently, the plates were washed three times with PBS Tween-20 (0.05%). They were then incubated with blocking buffer (consisting of 0.05% Tween-20 in PBS and 3% skimmed milk powder) for 1 h at 37 °C and washed with PBS Tween-20 (0.05%). Serum samples were diluted (1:100) in blocking buffer. Plates were incubated with the diluted chicken serum for 1 h at 37 °C and washed three times in PBS Tween-20 (0.05%).

Specific anti-*Campylobacter* IgY antibodies were detected with alkaline phosphatase conjugated to goat anti-chicken IgY (1:1000) (Serotec, Oxford, UK) diluted in blocking buffer, for 1 h at 37 °C. Plates were washed with PBS Tween-20 (0.05%) and incubated with 100 μl per well of *p*-nitrophenyl phosphate in the dark for 30 min at room temperature. The reaction was stopped by the addition of 100 μl 3 N sodium hydroxide to each well. Absorbance was determined using a microplate reader at 405 nm. To determine secretory IgA levels in the ileum, a 20 cm section of ileum was taken and flushed using 10 mL of PBS. The flushed fluid was then centrifuged for 10 min at 500 × *g* and supernatant frozen at −20 °C. The secretory IgA in the ileal flush was measured using an IgA Chicken ELISA Kit (ab157691, Abcam, Cambridge, UK).

### Statistical analyses

The impacts of treatment and time since treatment on the levels of Bu1^+^, CD4^+^, CD8a^+^ and CD8b^+^ cells were investigated using generalised linear modelling. Data were log transformed to ensure normality. Trends in the counts of *Campylobacter* cells in the jejunum, ileum and ceca through time in challenged bursectomised and control birds were investigated using generalised linear modelling. The structural equation modelling was undertaken using the lavaan^27^,^28^ packages. Analyses were undertaken in the R statistical programming language.

## Additional Information

**How to cite this article:** Lacharme-Lora, L. *et al*. B lymphocytes play a limited role in clearance of *Campylobacter jejuni* from the chicken intestinal tract. *Sci. Rep.* **7**, 45090; doi: 10.1038/srep45090 (2017).

**Publisher's note:** Springer Nature remains neutral with regard to jurisdictional claims in published maps and institutional affiliations.

## Supplementary Material

Supplementary Figure

## Figures and Tables

**Table 1:** Path and standardised path coefficients and significance for the Model 1 showing the relationship between bursectomised birds, IgY and *Campylobacter* load in the ileum, jejunum and caecum.

Regressions	Path Coefficient (SE)	Standardised path coefficient	*Z*-value	*P*-value
log IgY ~ treatment	−0.868 (0.054)	−0.913	−16.314	<0.001
log Ileum ~ log IgY	−7.936 (1.371)	−0.626	−5.786	<0.001
log Ileum ~ log Jejunum	0.551 (0.097)	0.621	5.713	<0.001
log Ileum ~ log Ceca	0.214 (0.057)	0.214	3.762	<0.001

The goodness of fit measures for the structural equation model were: Bollem-Stine p = 0.612, CFI = 0.999, RMSEA = 0.022, 90% Confidence Interval 0.000–0.195, SRMSR = 0.043.
