# Supplementary material for: B lymphocytes play a limited role in clearance of Campylobacter jejuni from the chicken intestinal tract
Source: Sci Rep. 2017 Mar 23;7:45090. doi: 10.1038/srep45090 (PMC5362810; doi:10.1038/srep45090)
Supplement: Supplementary Figure [file srep45090-s1.doc]

B lymphocytes play a limited role in clearance of *Campylobacter jejuni* from the chicken intestinal tract.

Lizeth Lacharme-Lora, Gemma Chaloner, Rachel Gilroy, Suzanne Humphrey, Kirsty Gibbs, Sue Jopson, Elli Wright, William Reid, Julian Ketley, Tom Humphrey, Nicola Williams, Steven Rushton, Paul Wigley

**Supplementary Information**

**Figure S1. Effect of bursectomy on B and T cell populations in the bursa and splenic tissues.** B cell depletion in young chicks was achieved by the chemical bursectomy method using cyclophosphamide. To assess the effect of the treatment on B and T cell populations, blood samples from birds were taken at 4 weeks of age. The Bu-1 marker was used to identify avian B lymphocytes (A, B); CD4, CD8α and CD8β markers were used for T lymphocytes(C-E). We observed a depletion of bursal and splenic B cell populations, while there was no evident effect of bursectomy on T cell population in the spleen. There were 6-7 birds per group. Bars represent the median values; circles represent individual birds. ** = p ≤0.01; NS= non-significant.

**
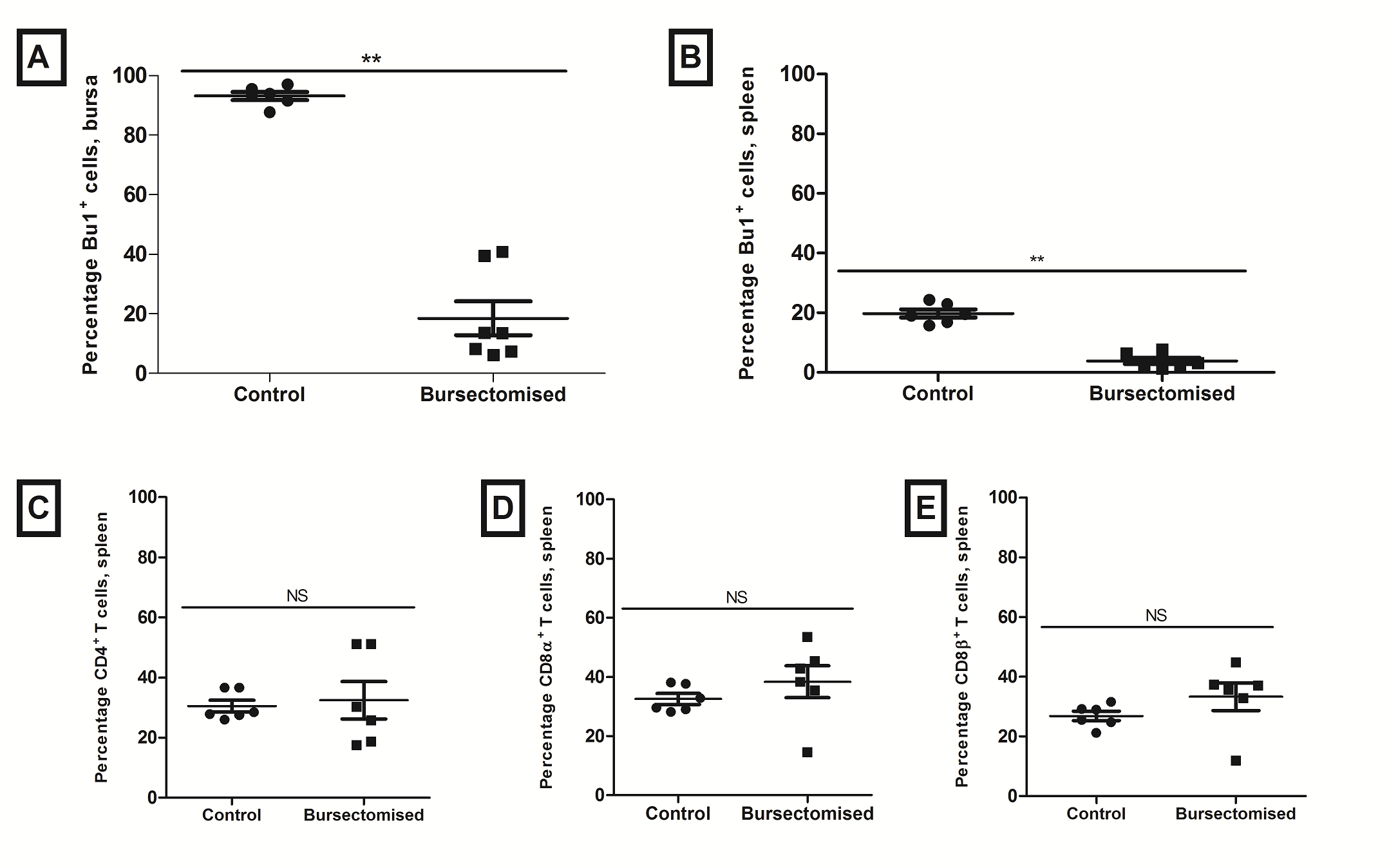
**
